# Supplementary material for: Virtual histological staining of unlabeled autopsy tissue
Source: Nat Commun. 2024 Feb 23;15:1684. doi: 10.1038/s41467-024-46077-2 (PMC10891155; doi:10.1038/s41467-024-46077-2)
Supplement: Supplementary file 1 — Supplementary Information [file 41467_2024_46077_MOESM1_ESM.pdf]

Supplementary Information for

Virtual histological staining of unlabeled autopsy tissue

Yuzhu Li[†,1,2,3], Nir Pillar[†,1,2,3], Jingxi Li[†,1,2,3], Tairan Liu[1,2,3], Di Wu[4], Songyu Sun[4], Guangdong Ma[1,5], Kevin de Haan[1,2,3], Luzhe Huang[1,2,3], Yijie Zhang[1,2,3], Sepehr Hamidi[6], Anatoly Urisman[7], Tal Keidar Haran[8], William Dean Wallace[9], Jonathan E. Zuckerman[6], and Aydogan Ozcan[*,1,2,3,10]

[1]Electrical and Computer Engineering Department, University of California, Los Angeles, CA, 90095, USA.

[2]Bioengineering Department, University of California, Los Angeles, 90095, USA.

[3]California NanoSystems Institute (CNSI), University of California, Los Angeles, CA, 90095, USA.

[4]Computer Science Department, University of California, Los Angeles, CA, 90095, USA.

[5]School of Physics, Xi'an Jiaotong University, Xi'an, 710049, China.

[6]Department of Pathology and Laboratory Medicine, David Geffen School of Medicine, University of California Los Angeles, Los Angeles, CA, 90095, USA.

[7]Department of Pathology, University of California, San Francisco, CA, 94143, USA.

[8]Department of Pathology, Hadassah Hebrew University Medical Center, Jerusalem, 91120, Israel.

[9]Department of Pathology, Keck School of Medicine, University of Southern California, Los Angeles, CA, 90033, USA.

[10]Department of Surgery, University of California, Los Angeles, CA, 90095, USA.

*Correspondence: Aydogan Ozcan Email: ozcan@ucla.edu

† Equal contributing authors

Supplementary Notes

Supplementary Note 1: Virtual staining performance comparison between RegiStain training framework and other network architectures

To further highlight the autopsy virtual staining performance of the RegiStain training framework and the critical role of using network R during the training process, we trained and blindly tested two other network architectures, including (1) a supervised GAN framework that employs a TransUNet as its virtual stain generator network, which characterizes a vision transformer-based structure to enhance the capture of long-range dependencies across an entire image; and (2) an unsupervised GAN framework (CycleGAN), which uses cycle-consistency loss to enable domain translation without the need for well-paired image data. Both of these frameworks used for comparison do not incorporate any fine registration mechanism (provided by network R in RegiStain) as a part of the training. The training/validation/testing datasets were kept the same among these three frameworks.

After the training, Supplementary Figure 8 provides an exemplary visualization of the virtually stained H&E images generated by these three frameworks, compared to their corresponding histochemically stained ground truth images obtained from *well-preserved* tissue regions. These results indicate that our RegiStain framework consistently provides virtual staining results superior to those from the other two frameworks, presenting decent structural and color correspondence with their histochemically stained ground truths. In contrast, the other two frameworks introduced severe staining errors on one or more test FOVs. For example, in Supplementary Figure 8f, the virtual staining results generated by the TransUNet-based GAN exhibited a notable failure in staining red blood cells. Moreover, the results in Supplementary Figure 8g-h also depict a marked missing of nuclear features. For the CycleGAN-based supervised framework, Supplementary Figure 8j similarly reveals strong artifacts in the staining of red blood cells. Furthermore, failures are apparent in the staining of nuclei, including both their spatial positions and size, as evidenced in Supplementary Figure 8i-l. These suboptimal staining results from the TransUNet-based GAN and CycleGAN frameworks corroborate our relevant analyses in the Discussion section of the main text: without effective supervised signals related to structural differences between the network prediction and the ground truth, which can be only attained by comparing precisely aligned paired image data, the optimal learning of the image transformation task is very hard to achieve, irrespective of deploying more complex and advanced image transformation architectures such as a TransUNet.

We also quantitatively evaluated these virtual staining results from different network architectures against their histochemical ground truth images using the same 100 test FOVs (8000 × 8000 pixels) used for generating Figs. 6-7 in the main text. As shown in Supplementary Figure 9a-b, our RegiStain framework offers the highest SSIM and PSNR values, showcasing more accurate virtual staining results compared to the TransUNet-based GAN and CycleGAN frameworks. The morphological feature quantification provided in Supplementary Figure 9c-d also showed that our results using the RegiStain framework reveal no statistically significant difference (P >0.05, using a two-tailed paired t-test) from the ground truth images in terms of the distribution of the number of cell nuclei per FOV and the average nuclei size. In contrast, the virtual staining results from the TransUNet-based GAN and CycleGAN frameworks individually demonstrated statistically significant differences compared to the ground truth. In summary,

these findings highlight the advantages of using the RegiStain framework on autopsy virtual staining tasks.

Supplementary Note 2: Threshold determination for the metrics used in the image feature-based staining artifact identification

Here we provide the details regarding how we determined the threshold for the metrics used for image feature-based staining artifact identification. In order to divide 2,000 FOVs from 10 testing whole slide images (WSIs) into well and poorly-stained areas with autolysis-induced artifacts, we devised two distinct metrics: (1) *the area percentage of stained nuclei within the tissue region*, used to identify regions with under-staining artifacts in nuclei; and (2) *the average intensity of adjacent cytoplasmic-extracellular regions*, used to detect regions exhibiting under-staining artifacts in the cytoplasmic-extracellular regions. To determine the threshold for the first metric, one board-certified pathologist labeled 50 FOVs with decent histochemical staining quality in nuclei (denoted as No. 1-50) and 50 FOVs with nuclei under-staining artifacts (denoted as No. 51-100). Supplementary Figure 12a shows that a threshold of 0.01 distinctly separates the two selected sets of FOVs (i.e., FOVs No. 1-50 with good staining quality and FOVs No. 51-100 with staining artifacts). To blindly validate the effectiveness of this threshold, we performed an assessment using an additional set of 50 FOVs with decent nuclei staining quality and 50 FOVs with under-stained nuclei, which were labeled by the same pathologist and never seen/used during the threshold tuning phase. The results shown in Supplementary Figure 12b indicate that the same threshold (0.01) can successfully distinguish the well-stained regions from poorly-stained regions with an accuracy of 100%.

The same approach was also used for determining the threshold of the 2nd metric, i.e., "*the average intensity of adjacent cytoplasmic-extracellular regions*". Supplementary Figure 12c shows that a threshold of 0.07 distinctly separates the two selected sets of FOVs (i.e., No. 1-50 with good cytoplasmic-extracellular staining and No. 51-100 with under-staining issues in cytoplasmic-extracellular regions). This threshold was then blindly validated on another 100 unseen FOVs (No. 1-50 with good cytoplasmic-extracellular staining and No. 51-100 with under-staining issues in cytoplasmic-extracellular regions), resulting in an accuracy of 98%, as shown in Supplementary Figure 12d. Note that all these 400 FOVs used for the metric threshold determination were not included in the subsequent image quality assessments conducted by pathologists, ensuring a completely blinded evaluation process.

For this threshold determination process, we did not consider using intensity normalization across different WSIs, including background normalization. These histochemically stained WSIs, despite being processed by two different pathology labs, were imaged using the same bright-field slide scanner (Leica Biosystems Aperio AT2), thereby possessing very similar image intensity distributions. To ensure that the lack of background normalization did not influence our division between well and poorly stained image sets, we conducted a comparative test by performing background normalization to all the 400 FOVs, followed by a re-assessment of the distributions of each metric. The background normalization that we used can be expressed as:

$$\left(I_{\mathrm{norm,r}}, I_{\mathrm{norm,g}}, I_{\mathrm{norm,b}}\right) = \left(I_{\mathrm{raw,r}}/\bar{r}, I_{\mathrm{raw,g}}/\bar{g}, I_{\mathrm{raw,b}}/b\right) \quad (1),$$

$$\left(\bar{r}, \bar{g}, \bar{b}\right) = \left(\frac{1}{mn}\sum_{i=1}^{m}\sum_{j=1}^{n} I_{\mathrm{bg,r}}(m,n), \frac{1}{mn}\sum_{i=1}^{m}\sum_{j=1}^{n} I_{\mathrm{bg,g}}(m,n), \frac{1}{mn}\sum_{i=1}^{m}\sum_{j=1}^{n} I_{\mathrm{bg,b}}(m,n)\right) \quad (2),$$

where $I_{\mathrm{bg,r}}$, $I_{\mathrm{bg,g}}$ and $I_{\mathrm{bg,b}}$ represent the red, green and blue channels of the background regions within a given FOV of histochemically stained tissue, respectively, all having a dimension of $m \times n$ pixels. Each of these background regions was selected by manually cropping an area on the background of the image FOV without tissue. With the normalization factors $\bar{r}$, $\bar{g}$, and $\bar{b}$ that are computed from the average of $I_{\mathrm{bg,r}}$, $I_{\mathrm{bg,g}}$ and $I_{\mathrm{bg,b}}$, respectively, the three color channels of the original FOV images $I_{\mathrm{raw,r}}$, $I_{\mathrm{raw,g}}$ and $I_{\mathrm{raw,b}}$ are normalized to $I_{\mathrm{norm,r}}$, $I_{\mathrm{norm,g}}$ and $I_{\mathrm{norm,b}}$, respectively.

After applying this background normalization to the same 400 image FOVs, the results are provided in Supplementary Figure 13a-d. It can be observed that, when compared to their counterparts reported in Supplementary Figure 12a-d without using normalization, the overall distributions of the metrics for the same FOVs with normalization remained largely consistent, with only minor shifts in the absolute values. Moreover, after fine-tuning the corresponding thresholds using the normalized images, we found that, for the metric "*the area percentage of stained nuclei within the tissue region*", the division among the well and poorly-stained FOVs in the validation set remained identical. Also, for the 2nd metric, "*the average intensity of adjacent cytoplasmic-extracellular regions*", the division using normalized images presented a small discordance of <5% in comparison to using the original images. These analyses reveal that the influence of background normalization on delineating between the well and poorly-stained histochemical FOVs is negligible. Considering the fact that the comparative analysis between the histochemically stained and virtually stained images would always be conducted in a paired fashion, we opted not to incorporate this normalization step during the final categorization of the 2,000 testing FOVs for evaluation purposes.

Supplementary Figures

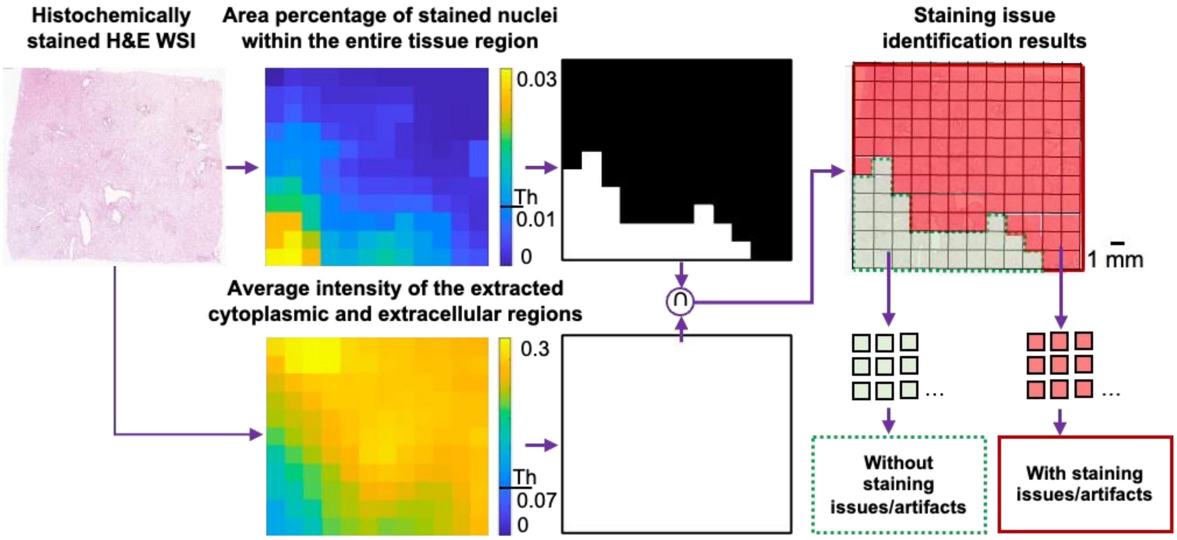

Supplementary Figure 1. Workflow for the image feature-based staining artifact identification. Each histochemically stained H&E slide is divided into smaller sample FOVs of 8000 × 8000 pixels. Each sample FOV is then quantified using two metrics: (1) the area percentage of the stained nuclei within the entire tissue region and (2) the average intensity of the extracted cytoplasmic and extracellular channels. These obtained values of the sample FOVs undergo a subsequent thresholding process, resulting in the sample FOVs identified as 'without staining artifacts' only if both of the metrics exceeded their respective thresholds, or classified as 'with staining artifacts' otherwise.

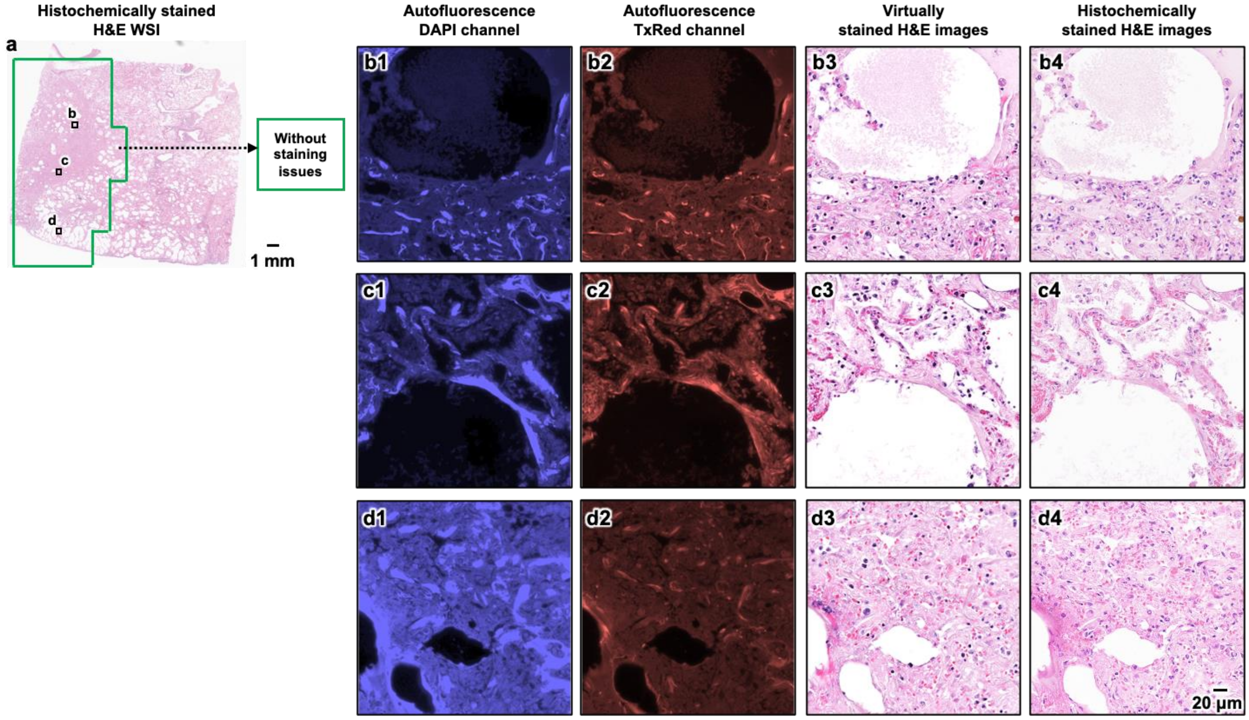

Supplementary Figure 2. Visual comparisons between the virtually stained H&E images of the same WSI used in Fig. 3 and their corresponding histochemical H&E images that exhibit decent staining quality (corresponding to well-preserved tissue regions). a, Histochemical H&E staining results of the WSI, which are identical to those in Fig. 3(c), main text. After the staining artifact quantification/identification process, the green-framed region is found to exhibit decent staining quality (corresponding to well-preserved tissue regions). **b-d,** Zoomed-in images of the three exemplary regions indicated in (a), which are selected from the areas exhibiting decent staining quality within the histochemically stained WSI in Fig. 3(c), main text. Here, (b1), (c1), and (d1) are the autofluorescence images of these regions captured using the DAPI channel, and (b2), (c2), and (d2) are their counterparts captured using the TxRed channel. These DAPI and TxRed autofluorescence image pairs serve as the inputs to our autopsy virtual staining network. (b3), (c3), and (d3) are the virtual H&E staining results corresponding to the same regions of (b1), (c1) and (d1) (or (b2), (c2) and (d2)), which are digitally generated by our virtual staining network based on (b1) and (b2), (c1) and (c2), and (d1) and (d2), respectively. (b4), (c4), and (d4) are the histochemical H&E staining results corresponding to the same regions of (b3), (c3), and (d3), which exhibit decent staining quality without staining issues, corresponding to well-preserved tissue regions. The process of producing these representative images in this figure was repeated, yielding similar results for all the 10 autopsy slides (n=10) in the blind testing stage.

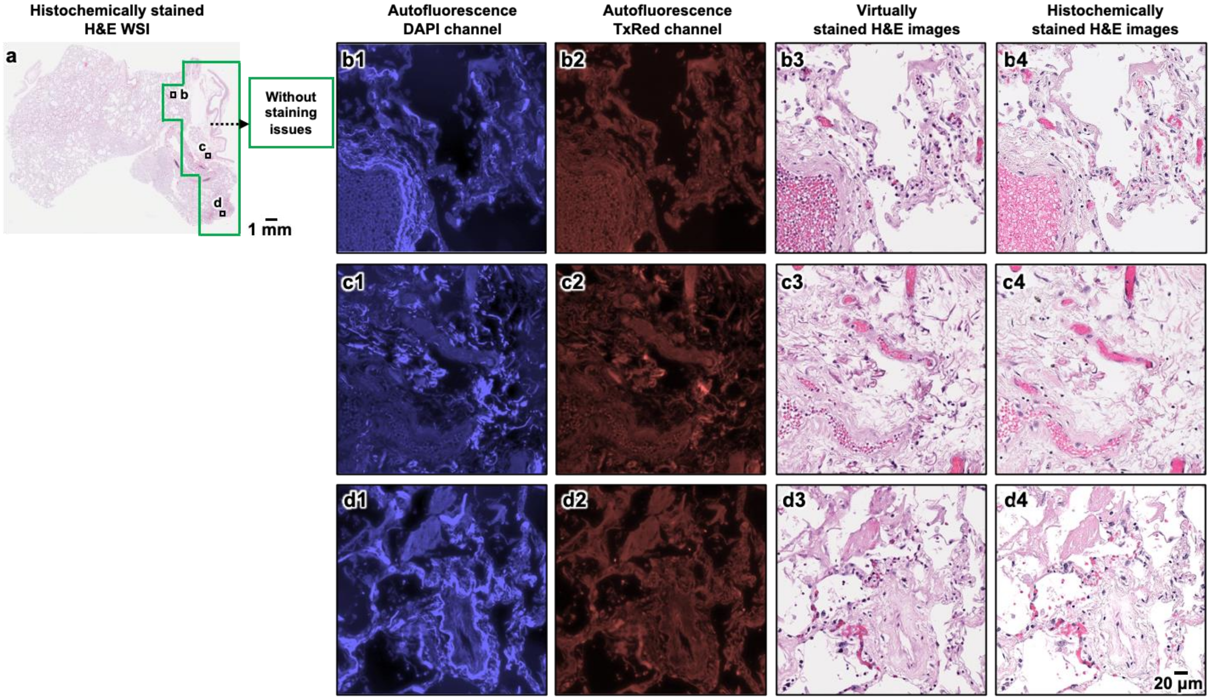

Supplementary Figure 3. Visual comparisons between the virtually stained H&E images of the same WSI used in Fig. 4 and their corresponding histochemical H&E images that exhibit decent staining quality (corresponding to well-preserved tissue regions). a, Histochemical H&E staining results of the WSI, which are identical to those in Fig. 4(c), main text. After the staining artifact quantification/identification process, the green-framed region is found to exhibit decent staining quality (corresponding to well-preserved tissue regions). **b-d,** Zoomed-in images of the three exemplary regions indicated in (a), which are selected from the areas exhibiting decent staining quality within the histochemically stained WSI in Fig. 4(c), main text. Here, (b1), (c1), and (d1) are the autofluorescence images of these regions captured using the DAPI channel, and (b2), (c2), and (d2) are their counterparts captured using the TxRed channel. These DAPI and TxRed autofluorescence image pairs serve as the inputs to our autopsy virtual staining network. (b3), (c3), and (d3) are the virtual H&E staining results corresponding to the same regions of (b1), (c1) and (d1) (or (b2), (c2) and (d2)), which are digitally generated by our virtual staining network based on (b1) and (b2), (c1) and (c2), and (d1) and (d2), respectively. (b4), (c4), and (d4) are the histochemical H&E staining results corresponding to the same regions of (b3), (c3), and (d3), which exhibit decent staining quality without staining issues, corresponding to well-preserved tissue regions. The process of producing these representative images in this figure was repeated, yielding similar results for all the 10 autopsy slides (n=10) in the blind testing stage.

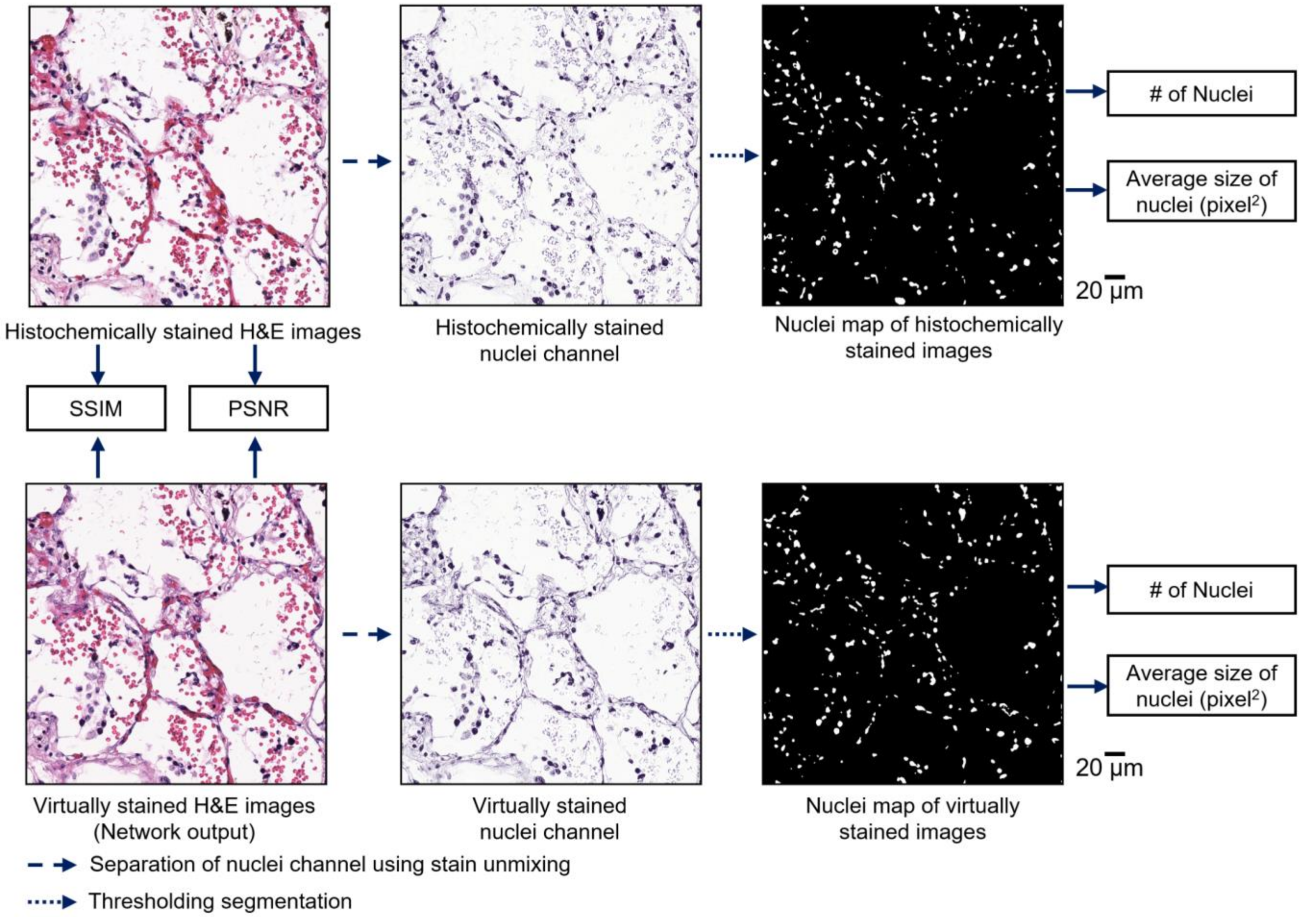

Supplementary Figure 4. Workflow for quantifying the SSIM, PSNR, the number of nuclei per FOV and the average nuclei size within the virtually and histochemically stained images. The details of the workflow can be found in the subsection "Algorithms used for quantitative evaluation of staining" of the Methods section in the main text.

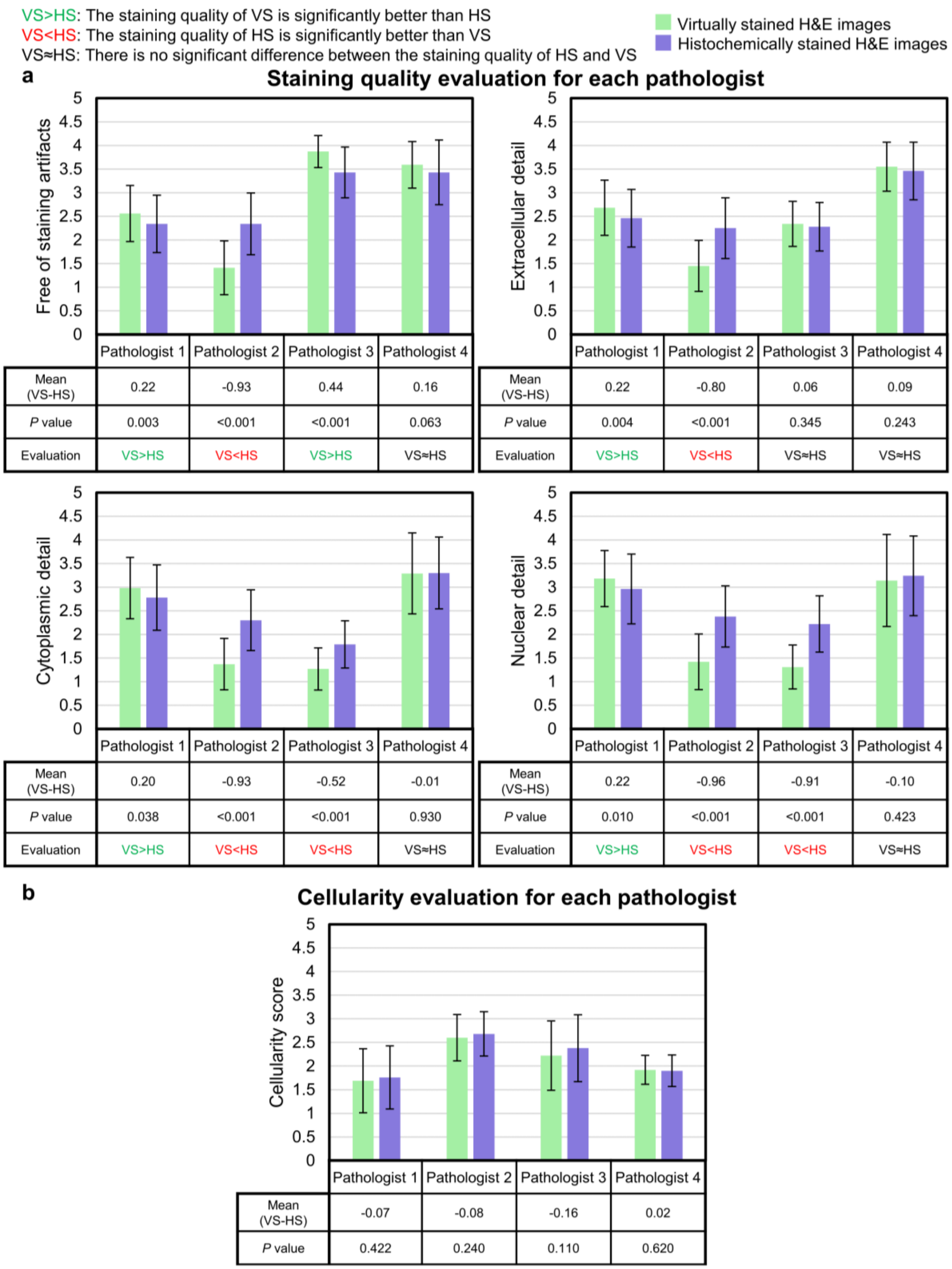

Free of staining artifacts:

	Pathologist 1	Pathologist 2	Pathologist 3	Pathologist 4
Mean (VS-HS)	0.22	-0.93	0.44	0.16
P value	0.003	<0.001	<0.001	0.063
Evaluation	VS>HS	VS<HS	VS>HS	VS≈HS

Extracellular detail:

	Pathologist 1	Pathologist 2	Pathologist 3	Pathologist 4
Mean (VS-HS)	0.22	-0.80	0.06	0.09
P value	0.004	<0.001	0.345	0.243
Evaluation	VS>HS	VS<HS	VS≈HS	VS≈HS

Cytoplasmic detail:

	Pathologist 1	Pathologist 2	Pathologist 3	Pathologist 4
Mean (VS-HS)	0.20	-0.93	-0.52	-0.01
P value	0.038	<0.001	<0.001	0.930
Evaluation	VS>HS	VS<HS	VS<HS	VS≈HS

Nuclear detail:

	Pathologist 1	Pathologist 2	Pathologist 3	Pathologist 4
Mean (VS-HS)	0.22	-0.96	-0.91	-0.10
P value	0.010	<0.001	<0.001	0.423
Evaluation	VS>HS	VS<HS	VS<HS	VS≈HS

Cellularity score:

	Pathologist 1	Pathologist 2	Pathologist 3	Pathologist 4
Mean (VS-HS)	-0.07	-0.08	-0.16	0.02
P value	0.422	0.240	0.110	0.620

Supplementary Figure 5. Same as Fig. 7, main text, except that the evaluation results by each of the four board-certified pathologists are shown individually. a, Staining quality scores of virtually and histochemically stained H&E images evaluated by each of the four board-certified pathologists, along with

the statistical evaluation results of the two-tailed paired t-test. The mean and standard deviation values of these scores for each metric and pathologist were calculated across all the 100 test sample FOVs (n=100). **b,** Cellularity scores evaluated by each of the four pathologists using all the 100 test sample FOVs (n=100), along with the statistical evaluation results of the two-tailed paired t-test.

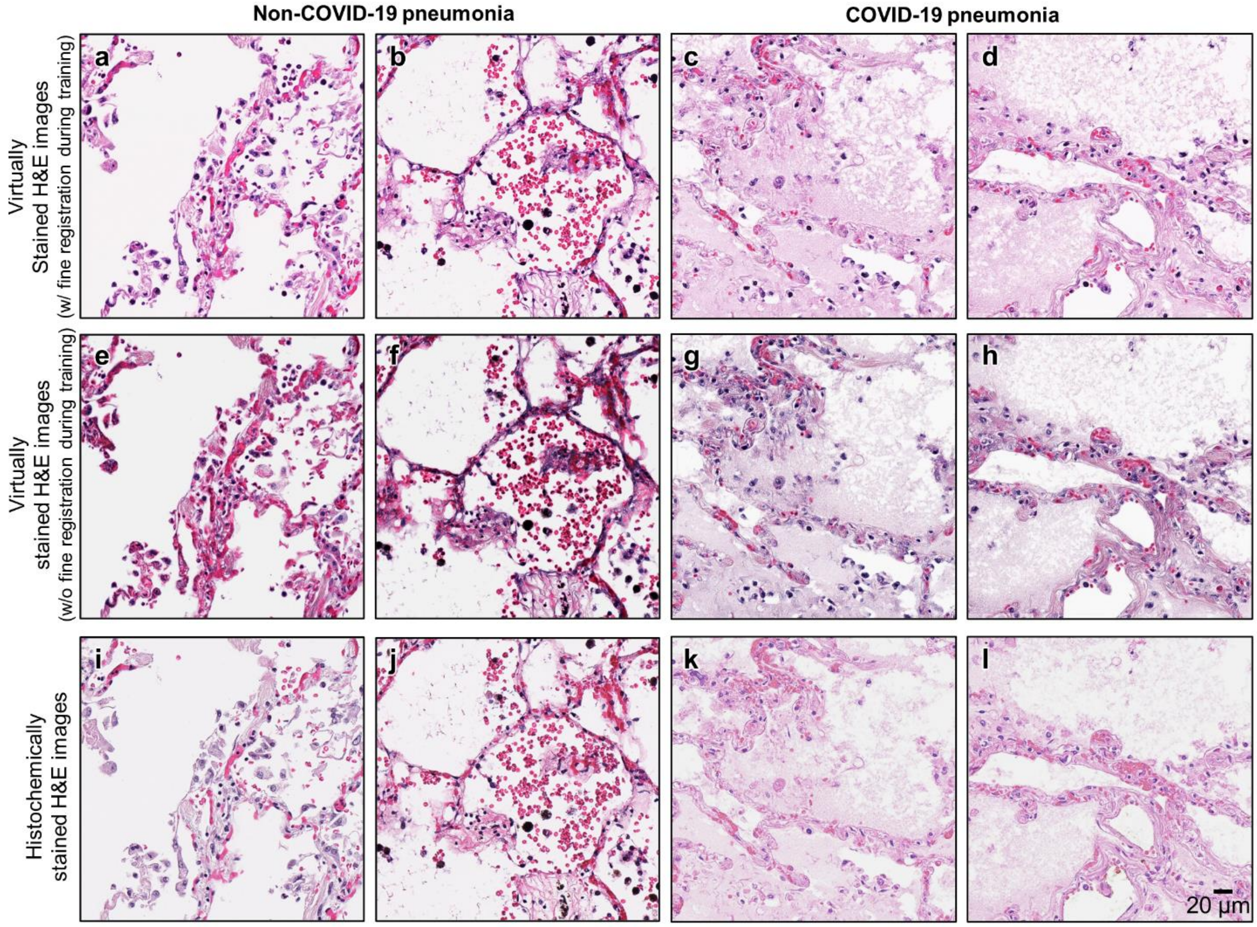

Supplementary Figure 6. Visual comparison of the virtually stained H&E images generated by the autopsy virtual staining models trained with and without the use of network R, along with their histochemically stained ground truth corresponding to well preserved tissue regions. a-d, Same as Fig. 5(i)-(l), main text, which show the virtually stained H&E images produced by the autopsy virtual staining model with the use of network R in the training framework. **e-h,** Virtually stained H&E images produced by the autopsy virtual staining model without the use of network R in the training framework. **i-l**, Same as Fig. 5(m)-(p), which show the histochemical H&E staining results of the same well-preserved tissue areas in (a)-(d) (or (e)-(h)). The process of producing these representative images in this figure was repeated, yielding similar results for all the 10 autopsy slides (n=10) in the blind testing stage.

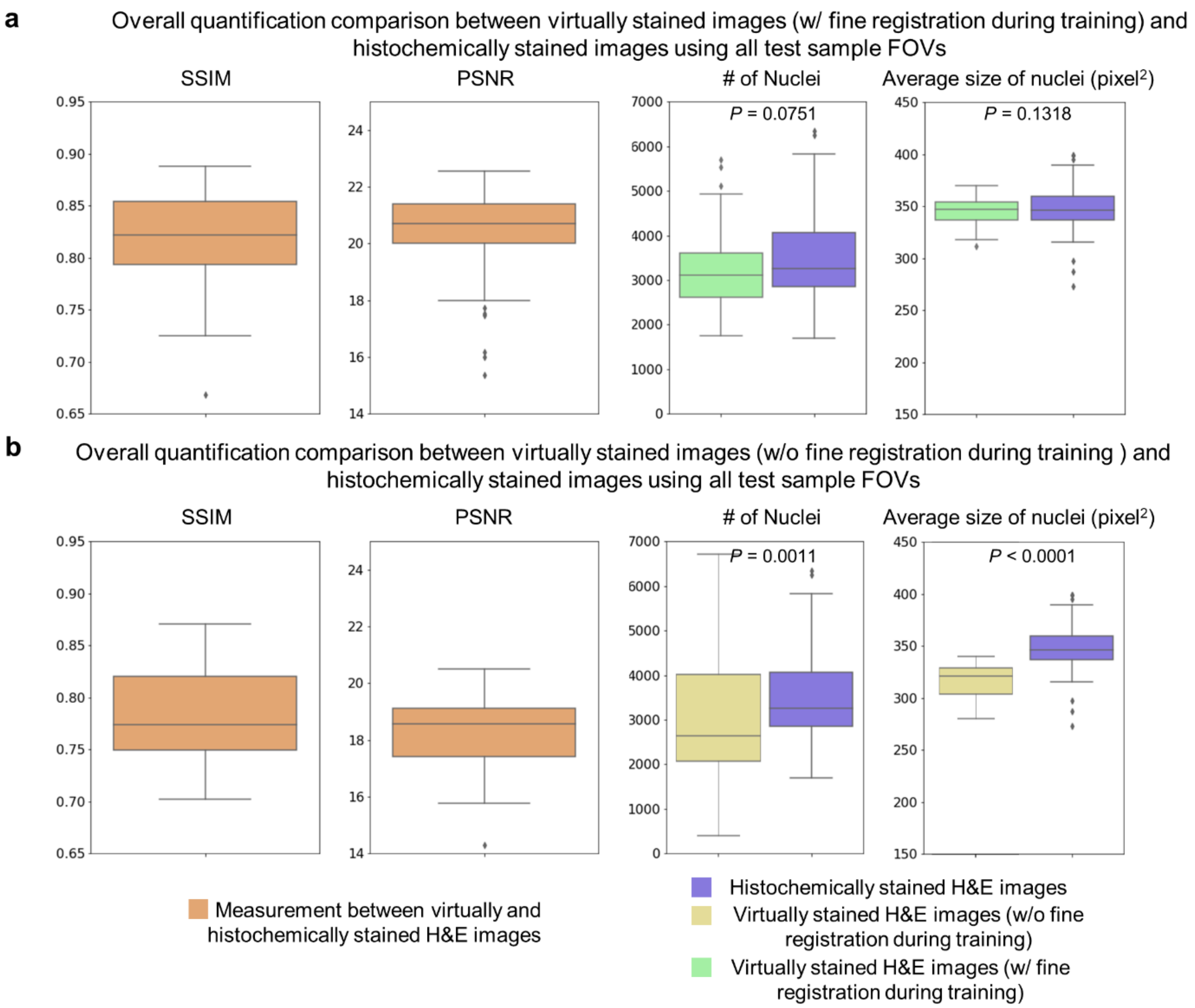

Supplementary Figure 7. Quantitative evaluation of the virtual H&E staining results generated by autopsy virtual staining models trained with and without the use of network R. a, Box plots showing the distributions of different metrics quantified using 100 test sample FOVs (n=100), where the virtually stained images were generated by the autopsy virtual staining model with fine registration during training (i.e., the network R was included in the training framework). **b,** Box plots showing the distributions of different metrics quantified using 100 test sample FOVs (n=100), where the virtually stained images used were generated by the autopsy virtual staining model without fine registration during training (i.e., the network R was removed from the training framework). For each box plot, the center is denoted by the median. The bounds of each box are defined by the lower quartile (25th percentile) and the upper quartile (75th percentile). The whiskers extend from the box and represent the data points that fall within 1.5 times the interquartile range from the lower and upper quartiles. Any data point outside this range is considered an outlier and plotted individually.

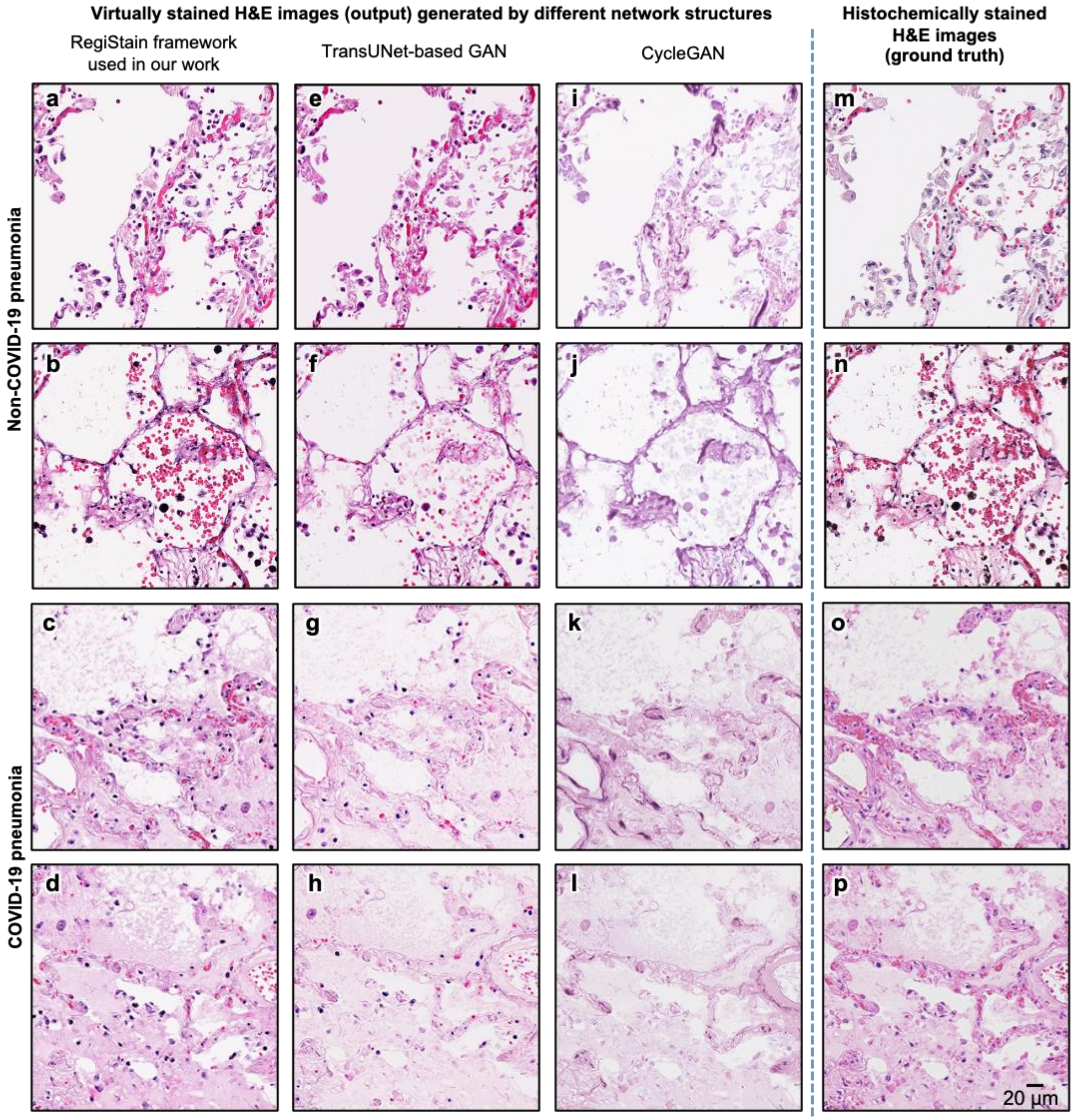

Supplementary Figure 8. Visual comparison of the virtually stained H&E images generated by different deep learning frameworks, along with their histochemically stained ground truth images obtained from well-preserved tissue regions. (a-d), (e-h) and (i-l) are the virtually stained H&E images produced by the RegiStain, TransUNet-based GAN and CycleGAN frameworks using the same four test FOVs, respectively, which are compared with their histochemically stained ground truth images (from well-preserved tissue regions) as shown in (m)-(p). The process of producing these representative images in this figure was repeated, yielding similar results for all the 10 autopsy slides (n=10) in the blind testing stage.

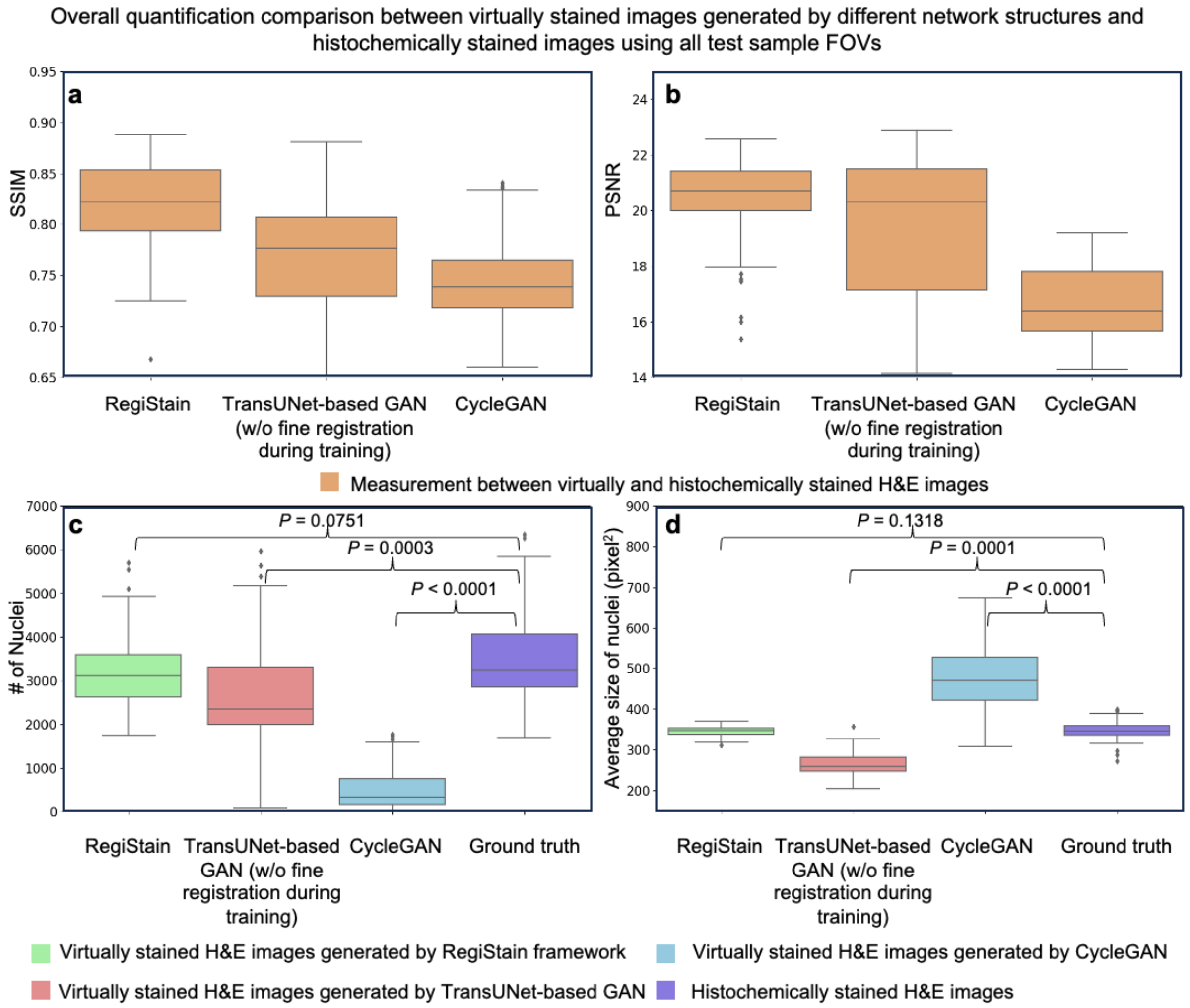

Supplementary Figure 9. Quantitative comparison of the virtual H&E staining results generated by different deep learning frameworks, including RegiStain, TransUNet-based GAN and CycleGAN. a, Box plots comparing the distributions of the SSIM values obtained from the virtual staining results of the different deep learning frameworks using 100 test sample FOVs (n=100). **b,** Same as (a), but the metric used is PSNR. **c,** Box plots comparing the distributions of the number of nuclei per FOV quantified on the virtual staining results of the different deep learning frameworks as well as their histochemically stained ground truth images using 100 test sample FOVs (n=100). Each *P* value was calculated by a two-tailed paired t-test. **d,** Box plots comparing the distributions of the average size of nuclei quantified on the virtual staining results of the different deep learning frameworks as well as their histochemically stained ground truth images using 100 test sample FOVs (n=100). Each *P* value was calculated by a two-tailed paired t-test. For each box plot, the center is denoted by the median. The bounds of each box are defined by the lower quartile (25th percentile) and the upper quartile (75th percentile). The whiskers extend from the box and represent the data points that fall within 1.5 times the interquartile range from the lower and upper quartiles. Any data point outside this range is considered an outlier and plotted individually.

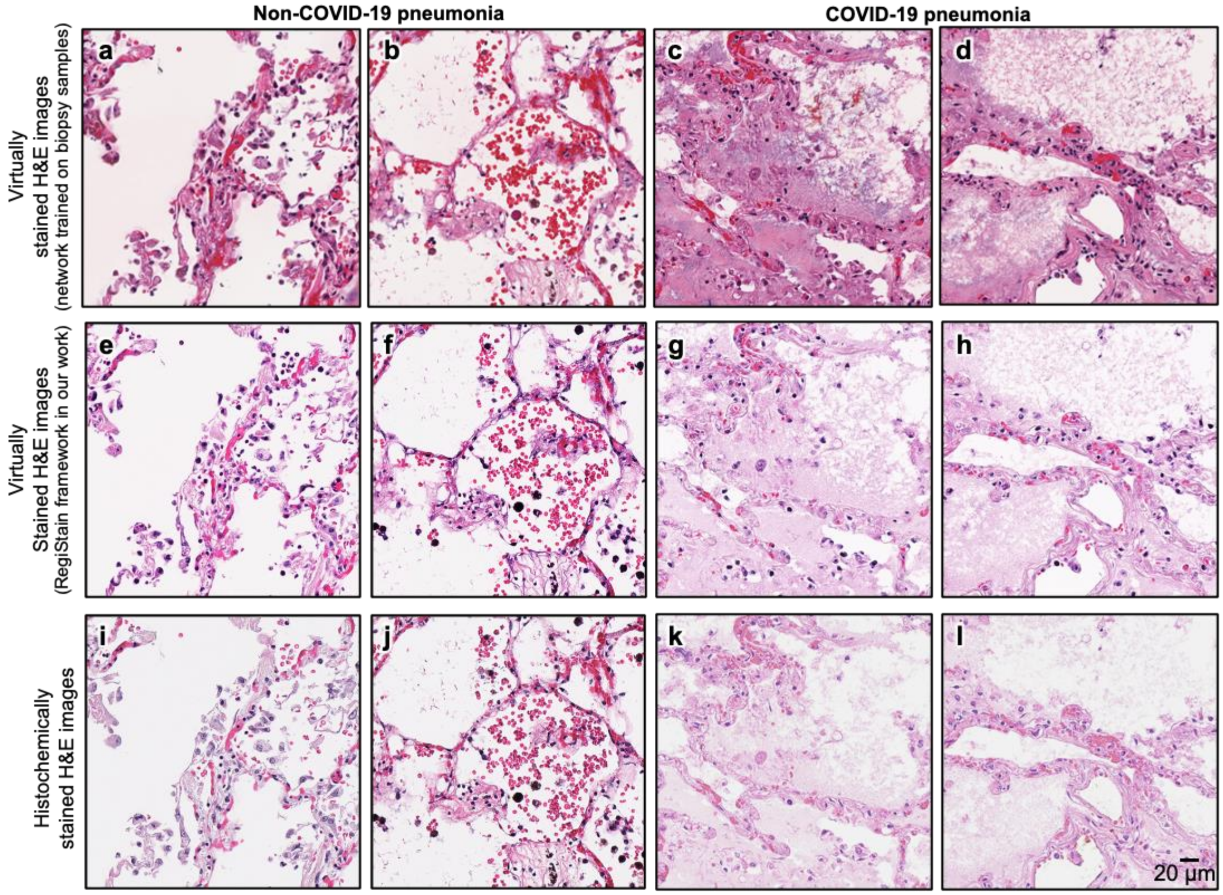

Supplementary Figure 10. Visual comparison of the virtually stained autopsy H&E images generated by an existing virtual staining model trained solely using lung biopsy data and our autopsy virtual staining model, along with their histochemically stained ground truth images corresponding to well-preserved tissue regions. a-d, Virtually stained autopsy H&E images produced by an established virtual staining model trained on lung biopsy data. **e-h,** Same as Figs. 5(i)-(l) of the main text, which show the virtually stained H&E images produced by our autopsy virtual staining model. **i-l**, Same as Figs. 5(m)-(p) of the main text, which show the histochemical H&E staining results of the same well-preserved tissue areas. The process of producing these representative images in this figure was repeated, yielding similar results for all the 10 autopsy slides (n=10) in the blind testing stage.

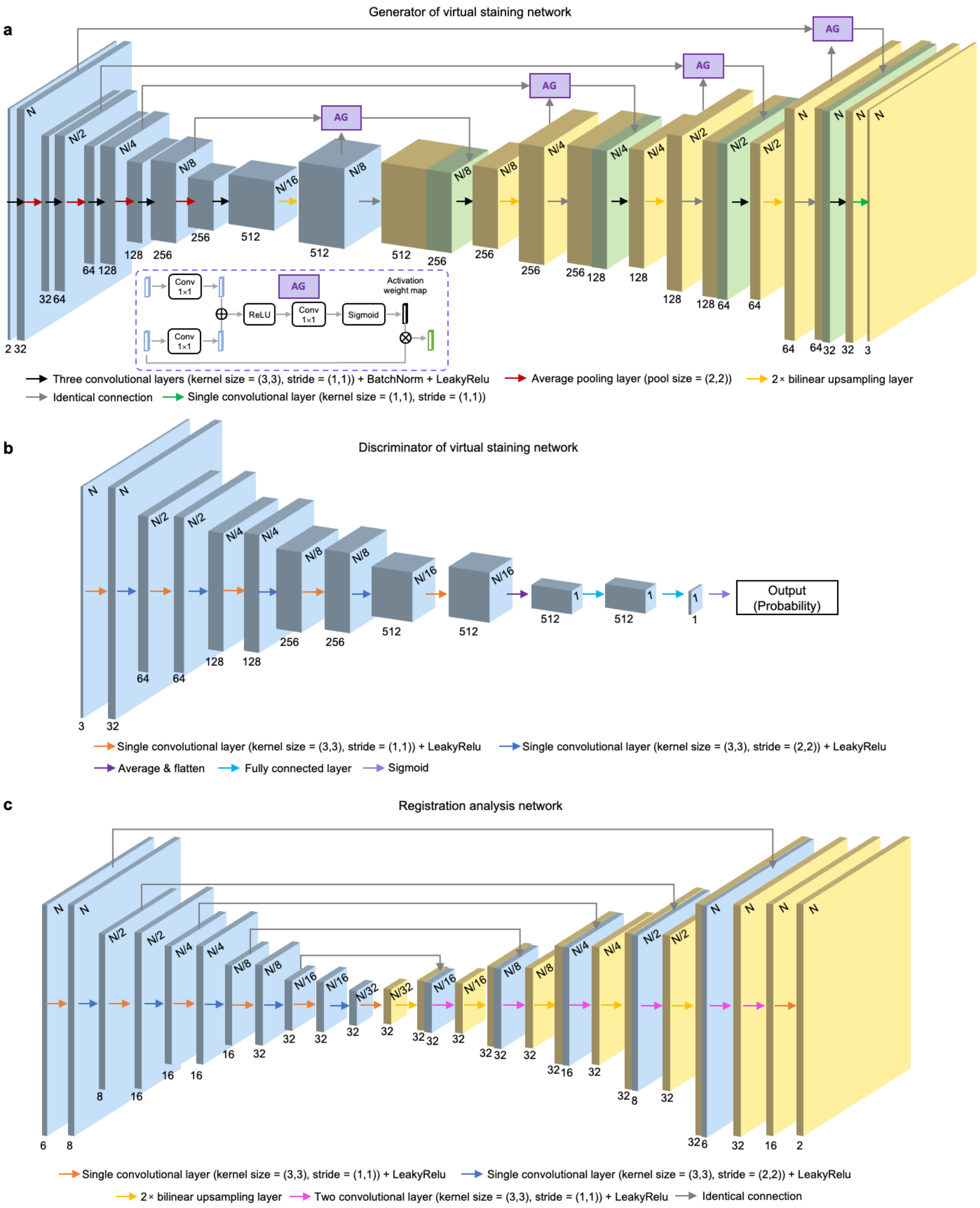

Supplementary Figure 11. Architectures of the networks used in the RegiStain framework. From (a) to (c), the virtual staining generator network (G), the discriminator network (D), and the registration analysis

network (R) are illustrated. More information can be found in the subsection "RegiStain framework and network architecture" of the Methods section in the main text.

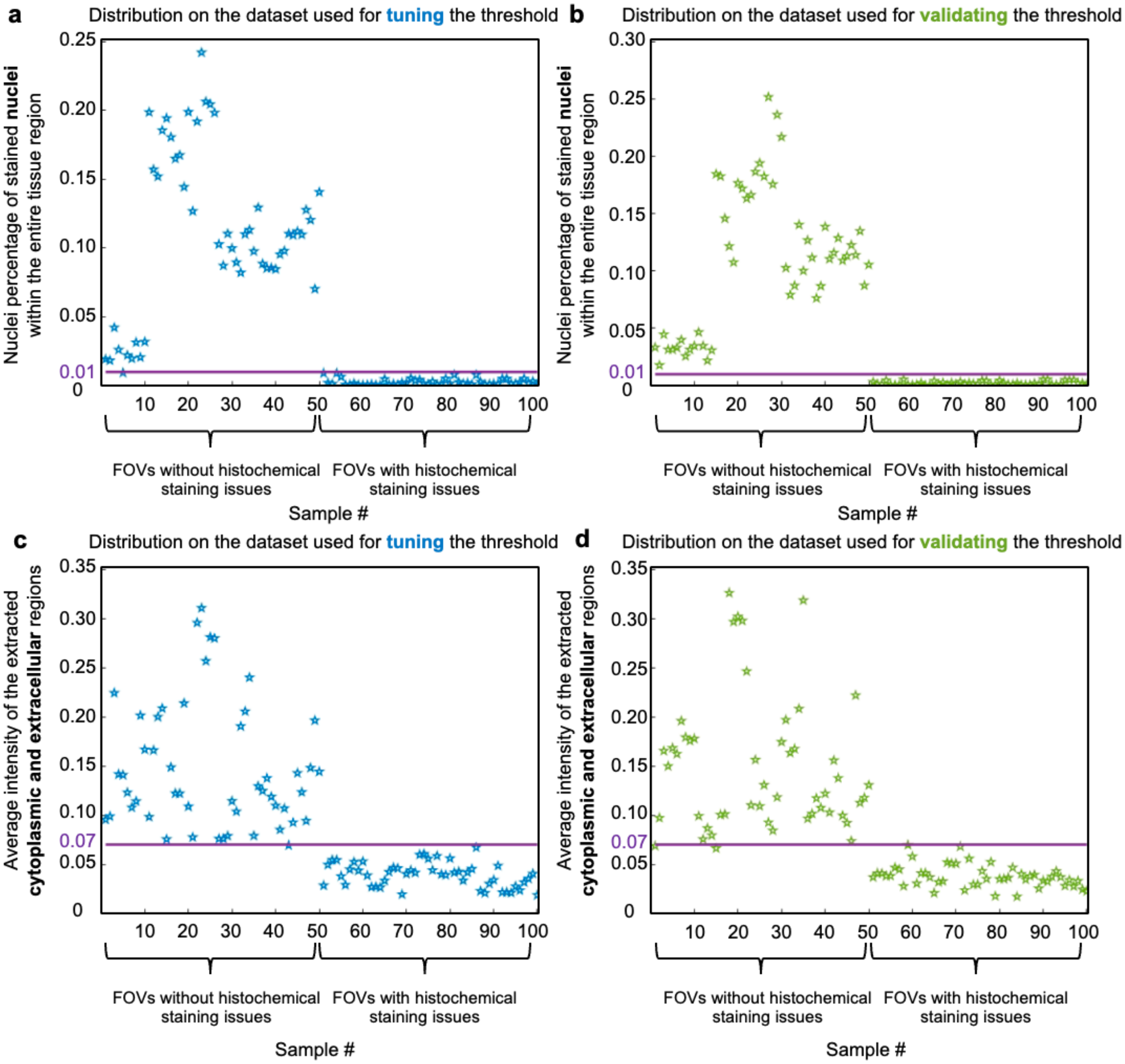

Supplementary Figure 12. Distributions of the metrics used in image feature-based staining artifact identification to differentiate the well-stained test sample FOVs before normalization. a, Distribution of the metric "*the area percentage of stained nuclei within the tissue region*" quantified from the 100 FOVs (n=100) used for tuning this threshold, where FOVs No. 1-50 have good nuclei staining and FOVs No. 51-100 have under-staining issues in nuclei regions. A threshold of 0.01 was selected for this metric. **b,** Similar to (a), but the same threshold is blindly tested on another 100 FOVs (n=100) different from (a). **c,** Similar to (a), but the metric here is "*the average intensity of adjacent cytoplasmic-extracellular regions*", which was quantified from another 100 FOVs (n=100), different from (a-b). A threshold of 0.07 was selected for this metric. **d,** Similar to (c), but the same threshold is blindly tested on another 100 FOVs (n=100) different from (a-c), used for validating the threshold determined from (c).

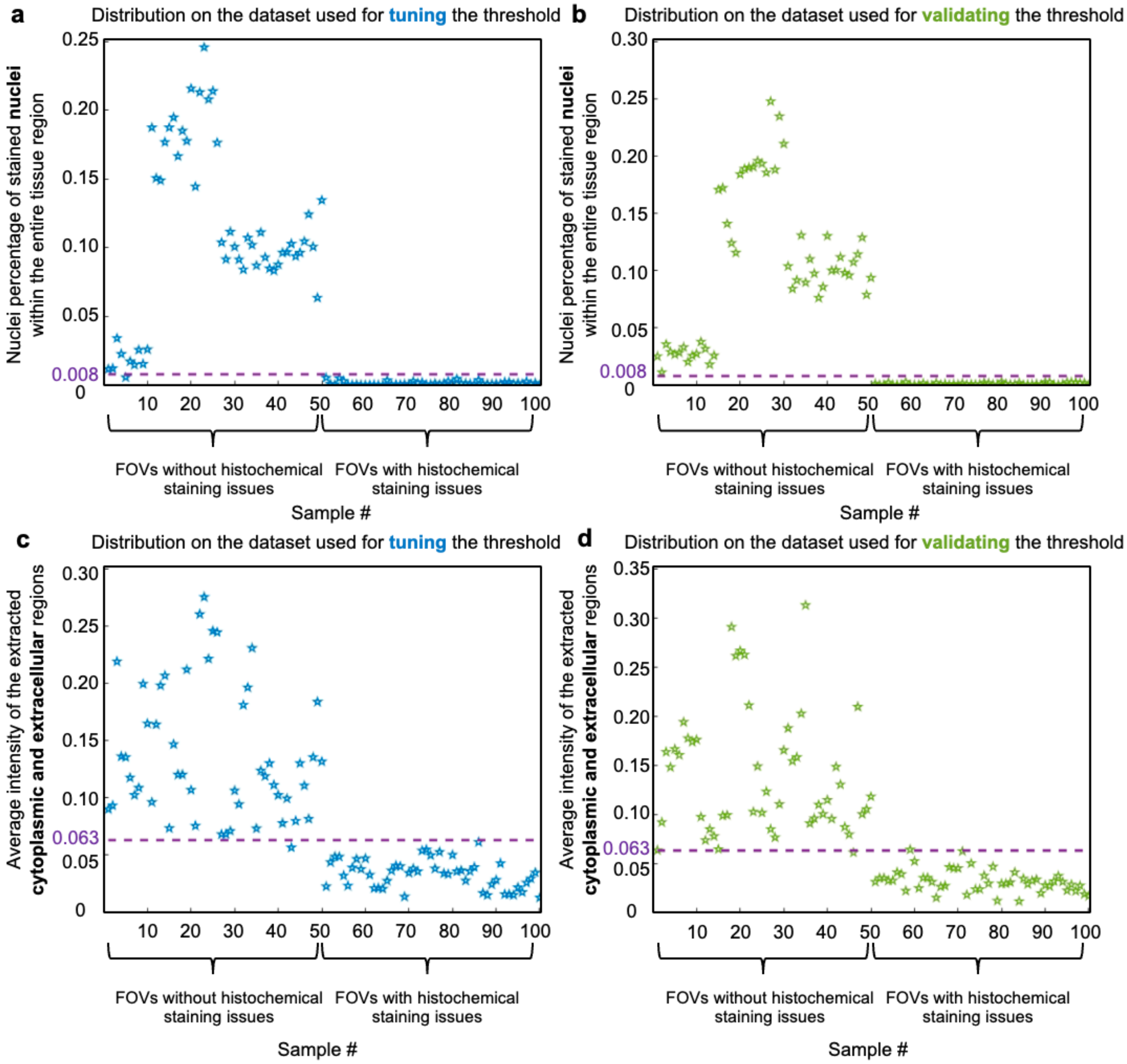

Supplementary Figure 13. Distributions of the metrics used in image feature-based staining artifact identification to differentiate the well-stained test sample FOVs after normalization. a, Distribution of the metric "the area percentage of stained nuclei within the tissue region" quantified from the 100 FOVs (n=100) used for tuning this threshold, where FOVs No. 1-50 have good nuclei staining and FOVs No. 51-100 have under-staining issues in nuclei regions. A threshold of 0.008 was selected for this metric. **b,** Similar to (a), but the same threshold is blindly tested on another 100 FOVs (n=100) different from (a). **c,** Similar to (a), but the metric here is "the average intensity of adjacent cytoplasmic-extracellular regions", which was quantified from another 100 FOVs (n=100), different from (a-b). A threshold of 0.063 was selected for this metric. **d,** Similar to (c), but the same threshold is blindly tested on another 100 FOVs (n=100) different from (a-c), used for validating the threshold determined from (c).

Supplementary Table

Sample ID	Sample types	Diagnosis	COVID-19 condition	Elapsed time from death to autopsy	Percentage of area with staining issues	Comments
1	lung	pneumonia	Non-COVID-19	1 day	<1%	
2	lung	pneumonia	Non-COVID-19	2 days	<1%	
3	lung	pneumonia	Non-COVID-19	5 days	<1%	
4	lung	pneumonia	Non-COVID-19	1 day	<1%	
5	lung	pneumonia	Non-COVID-19	2 days	1.43%	
6	lung	pneumonia	Non-COVID-19	2 days	1.78%	
7	lung	pneumonia	Non-COVID-19	4 days	58.82%	Exemplary slide used in Fig. 4
8	lung	pneumonia	COVID-19	36 days	62.38%	Exemplary slide used in Fig. 3
9	lung	pneumonia	COVID-19	10 days	59.66%	
10	lung	pneumonia	COVID-19	15 days	76.92%	

Supplementary Table 1. Detailed information of the unique autopsy slides used for testing.
