# Supplementary material for: Virtual histological staining of unlabeled autopsy tissue
Source: Nat Commun. 2024 Feb 23;15:1684. doi: 10.1038/s41467-024-46077-2 (PMC10891155; doi:10.1038/s41467-024-46077-2)
Supplement: Supplementary file 3 — Reporting Summary [file 41467_2024_46077_MOESM3_ESM.pdf]

Corresponding author(s): Aydogan Ozcan

Last updated by author(s): Feb 3, 2024

Reporting Summary

Nature Portfolio wishes to improve the reproducibility of the work that we publish. This form provides structure for consistency and transparency in reporting. For further information on Nature Portfolio policies, see our Editorial Policies and the Editorial Policy Checklist.

Statistics

For all statistical analyses, confirm that the following items are present in the figure legend, table legend, main text, or Methods section.

n/a	Confirmed	
☐	☒	The exact sample size (*n*) for each experimental group/condition, given as a discrete number and unit of measurement
☐	☒	A statement on whether measurements were taken from distinct samples or whether the same sample was measured repeatedly
☐	☒	The statistical test(s) used AND whether they are one- or two-sided<br>*Only common tests should be described solely by name; describe more complex techniques in the Methods section.*
☒	☐	A description of all covariates tested
☒	☐	A description of any assumptions or corrections, such as tests of normality and adjustment for multiple comparisons
☐	☒	A full description of the statistical parameters including central tendency (e.g. means) or other basic estimates (e.g. regression coefficient) AND variation (e.g. standard deviation) or associated estimates of uncertainty (e.g. confidence intervals)
☐	☒	For null hypothesis testing, the test statistic (e.g. *F*, *t*, *r*) with confidence intervals, effect sizes, degrees of freedom and *P* value noted<br>*Give P values as exact values whenever suitable.*
☒	☐	For Bayesian analysis, information on the choice of priors and Markov chain Monte Carlo settings
☒	☐	For hierarchical and complex designs, identification of the appropriate level for tests and full reporting of outcomes
☒	☐	Estimates of effect sizes (e.g. Cohen's *d*, Pearson's *r*), indicating how they were calculated

Our web collection on statistics for biologists contains articles on many of the points above.

Software and code

Policy information about availability of computer code

Data collection	The autofluorescence images of unlabeled autopsy tissue sections were captured using a Leica DMI8 microscope, which was controlled by Leica LAS X microscopy automation software. The stained images were digitized using a brightfield slide scanner (Leica Biosystems Aperio AT2).
Data analysis	All the image processing and dataset generation were performed using MATLAB version R2022b (Mathworks). The neural networks were implemented and trained using Python 3.8.15 and TensorFlow 2.5.0. Statistical analyses were performed using IBM SPSS Statistics v29.0.

For manuscripts utilizing custom algorithms or software that are central to the research but not yet described in published literature, software must be made available to editors and reviewers. We strongly encourage code deposition in a community repository (e.g. GitHub). See the Nature Portfolio guidelines for submitting code & software for further information.

Data

Policy information about availability of data

All manuscripts must include a data availability statement. This statement should provide the following information, where applicable:

- Accession codes, unique identifiers, or web links for publicly available datasets
- A description of any restrictions on data availability
- For clinical datasets or third party data, please ensure that the statement adheres to our policy

All data supporting the results of this study are available withn the main text and Supplementary Information. Example testing images are provided at:

https://doi.org/10.5281/zenodo.10203424. Whole tissue slides corresponding to autopsy specimen were obtained under UCLA IRB 18-001029 from UCLA Health for the current study.

Research involving human participants, their data, or biological material

Policy information about studies with human participants or human data. See also policy information about sex, gender (identity/presentation), and sexual orientation and race, ethnicity and racism.

Reporting on sex and gender	Since the autopsy slides data that we used for this study were all de-identified and obtained from the UCLA Translational Pathology Core Laboratory (TPCL) in compliance with the ethical standards of UCLA Institutional Review Board (approval granted by UCLA IRB 18-001029), we are unable to provide the information such as sex, gender, race, ethnicity, and other aspects.
Reporting on race, ethnicity, or other socially relevant groupings	Since the autopsy slides data that we used for this study were all de-identified and obtained from the UCLA Translational Pathology Core Laboratory (TPCL) in compliance with the ethical standards of UCLA Institutional Review Board (approval granted by UCLA IRB 18-001029), we are unable to provide the information such as sex, gender, race, ethnicity, and other aspects.
Population characteristics	N/A
Recruitment	N/A
Ethics oversight	Autopsy tissue sections used for this study were sourced from existing deidentified specimens, collected before this work, from the UCLA Translational Pathology Core Laboratory (TPCL) under UCLA IRB 18-001029.

Note that full information on the approval of the study protocol must also be provided in the manuscript.

Field-specific reporting

Please select the one below that is the best fit for your research. If you are not sure, read the appropriate sections before making your selection.

☒ Life sciences ☐ Behavioural & social sciences ☐ Ecological, evolutionary & environmental sciences

For a reference copy of the document with all sections, see nature.com/documents/nr-reporting-summary-flat.pdf

Life sciences study design

All studies must disclose on these points even when the disclosure is negative.

Sample size	18 autopsy sample cases (including 3 COVID-19-induced pneumonia cases and 15 non-COVID-19-induced pneumonia cases) coming from unique patients were acquired.
Data exclusions	We did not exclude any data during the analysis.
Replication	We reproduced the technique using the 18 subjects described in the paper.
Randomization	3 cases corresponding to COVID-19-induced pneumonia were randomly selected by a board-certified pathologist. 15 cases corresponding to non-COVID-19-induced pneumonia were randomly selected by a board-certified pathologist. All training, validation, and testing samples were randomly chosen and allocated.
Blinding	All the evaluation of virtual staining results produced by the deep neural network was blindly performed on tissue images that were not included in the training or validation phases. During the assessment of the staining quality, the four board-certified pathologists were blind to information regarding whether each image was created through the virtual staining technique or the conventional histochemical staining process. Also, the evaluation results of each pathologist were kept confidential from the other pathologists.

Reporting for specific materials, systems and methods

We require information from authors about some types of materials, experimental systems and methods used in many studies. Here, indicate whether each material, system or method listed is relevant to your study. If you are not sure if a list item applies to your research, read the appropriate section before selecting a response.

Materials & experimental systems

n/a	Involved in the study
☒	☐ Antibodies
☒	☐ Eukaryotic cell lines
☒	☐ Palaeontology and archaeology
☒	☐ Animals and other organisms
☒	☐ Clinical data
☒	☐ Dual use research of concern
☒	☐ Plants

Methods

n/a	Involved in the study
☒	☐ ChIP-seq
☒	☐ Flow cytometry
☒	☐ MRI-based neuroimaging
